# ESR Essentials: using the right scoring system in prostate MRI—practice recommendations by ESUR

**DOI:** 10.1007/s00330-024-10792-7

**Published:** 2024-05-23

**Authors:** Andrea Ponsiglione, Giorgio Brembilla, Renato Cuocolo, Patricia Gutierrez, Ana Sofia Moreira, Martina Pecoraro, Jeries Zawaideh, Jelle Barentsz, Francesco Giganti, Anwar R. Padhani, Valeria Panebianco, Philippe Puech, Geert Villeirs

**Affiliations:** 1 Department of Advanced Biomedical Sciences, University of Naples Federico II, Naples, Italy; 2 Department of Radiology, IRCCS San Raffaele Scientific Institute, Vita-Salute San Raffaele University, Milan, Italy; 3 Department of Medicine, Surgery and Dentistry, University of Salerno, Baronissi, Italy; 4 Department of Radiology, CH Dunkerque, Dunkirk, France; 5 Department of Radiology, Centro Hospitalar Universitário do Algarve, Unidade de Faro, Faro, Portugal; 6 Department of Radiological Sciences, Oncology and Pathology, Sapienza University of Rome, Rome, Italy; 7 Department of Radiology, IRCCS Ospedale Policlinico San Martino, Genoa, Italy; 8 Imaging Department Andros Clinics, Arnhem, The Netherlands; 9 Department of Radiology, University College London Hospital NHS Foundation Trust, London, UK; 10 Division of Surgery and Interventional Science, University College London, London, UK; 11 Paul Strickland Scanner Centre, Mount Vernon Cancer Centre, Northwood, UK; 12 Department of radiology, U1189 - ONCO-THAI - Image Assisted Laser Therapy for Oncology, University of Lille Inserm, CHU Lille, Lille, France; 13 Department of Medical Imaging, Ghent University Hospital, Ghent, Belgium

**Keywords:** Prostate cancer, Magnetic resonance imaging, Classification; management, Standardization

## Abstract

**Abstract:**

MRI has gained prominence in the diagnostic workup of prostate cancer (PCa) patients, with the Prostate Imaging Reporting and Data System (PI-RADS) being widely used for cancer detection. Beyond PI-RADS, other MRI-based scoring tools have emerged to address broader aspects within the PCa domain. However, the multitude of available MRI-based grading systems has led to inconsistencies in their application within clinical workflows. The Prostate Cancer Radiological Estimation of Change in Sequential Evaluation (PRECISE) assesses the likelihood of clinically significant radiological changes of PCa during active surveillance, and the Prostate Imaging for Local Recurrence Reporting (PI-RR) scoring system evaluates the risk of local recurrence after whole-gland therapies with curative intent. Underlying any system is the requirement to assess image quality using the Prostate Imaging Quality Scoring System (PI-QUAL). This article offers practicing radiologists a comprehensive overview of currently available scoring systems with clinical evidence supporting their use for managing PCa patients to enhance consistency in interpretation and facilitate effective communication with referring clinicians.

**Key Points:**

- *Assessing image quality is essential for all prostate MRI interpretations and the PI-QUAL score represents  the standardized tool for this purpose*.
- *Current urological clinical guidelines for prostate cancer diagnosis and localization recommend adhering to the PI-RADS recommendations*.
- *The PRECISE and PI-RR scoring systems can be used for assessing radiological changes of prostate cancer during active surveillance and the likelihood of local recurrence after radical treatments respectively*.

## Key recommendations

- Image quality assessment is crucial before all MRI interpretations. Uniform reporting of image quality is essential, and the PI-QUAL score is the standardized tool for this purpose (Level of evidence: moderate).
- The use of prostate MRI before biopsy is recommended as a triage test in selected patients with clinical suspicion of prostate cancer and should be acquired and interpreted using PI-RADS v2.1 recommendations (Level of evidence: high).
- PRECISE assessments are designed to be used to predict the likelihood of clinically significant radiological changes during active surveillance of prostate cancer, whereas the PI-RR scoring system estimates the likelihood of local recurrence following primary whole-gland curative treatments (Level of evidence: moderate).

## Introduction

Prostate MRI plays a central role in prostate cancer (PCa) management with multiple applications in patient’s journey [1]. The Prostate Imaging Reporting and Data System (PI-RADS) is widely used for cancer detection and localization [2]. Besides PI-RADS, other MRI-based scoring tools have been proposed for different clinical situations. These systems find application: (i) in the evaluation of image quality [3]; (ii) to assess the likelihood of clinically significant radiological changes on serial imaging during active surveillance (AS) [4]; and (iii) to assess the risk of local recurrence after primary whole-gland treatments (i.e., radical surgery or radiotherapy) [5].

However, there is still confusion regarding the application of the appropriate scoring system and its timing within the clinical workflow (e.g., the incorrect application of PI-RADS in the post-treatment setting). Utilizing a scoring system is crucial to ensure consistency in radiological reports, promote standardized interpretations, and facilitate effective communication with physicians.

**Fig. 1:**
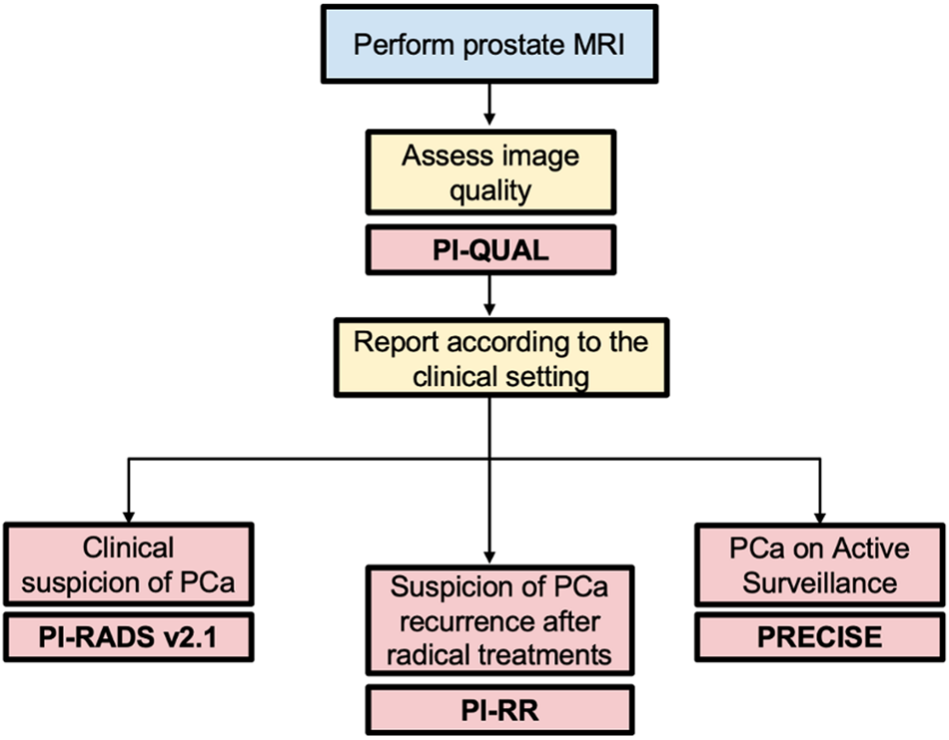
General flowchart of the available prostate MRI-based scoring systems according to clinical indications. MRI, magnetic resonance imaging; PI-QUAL, Prostate Imaging Quality; PCa, prostate cancer; PI-RADS, Prostate Imaging Reporting and Data System; PI-RR, Prostate Imaging for Local Recurrence Reporting; PRECISE, Prostate Cancer Radiological Estimation of Change in Sequential Evaluation

This paper provides a comprehensive overview of the main tools for prostate MRI, while also drawing attention to current evidence and limitations. Additionally, we present clear and concise flowcharts that can guide the general radiologist through the decision-making process, allowing the application of the relevant scoring system based on different clinical scenarios (Fig. 1).

## Prostate Imaging Reporting and Data System (PI-RADS)

**Fig. 2:**
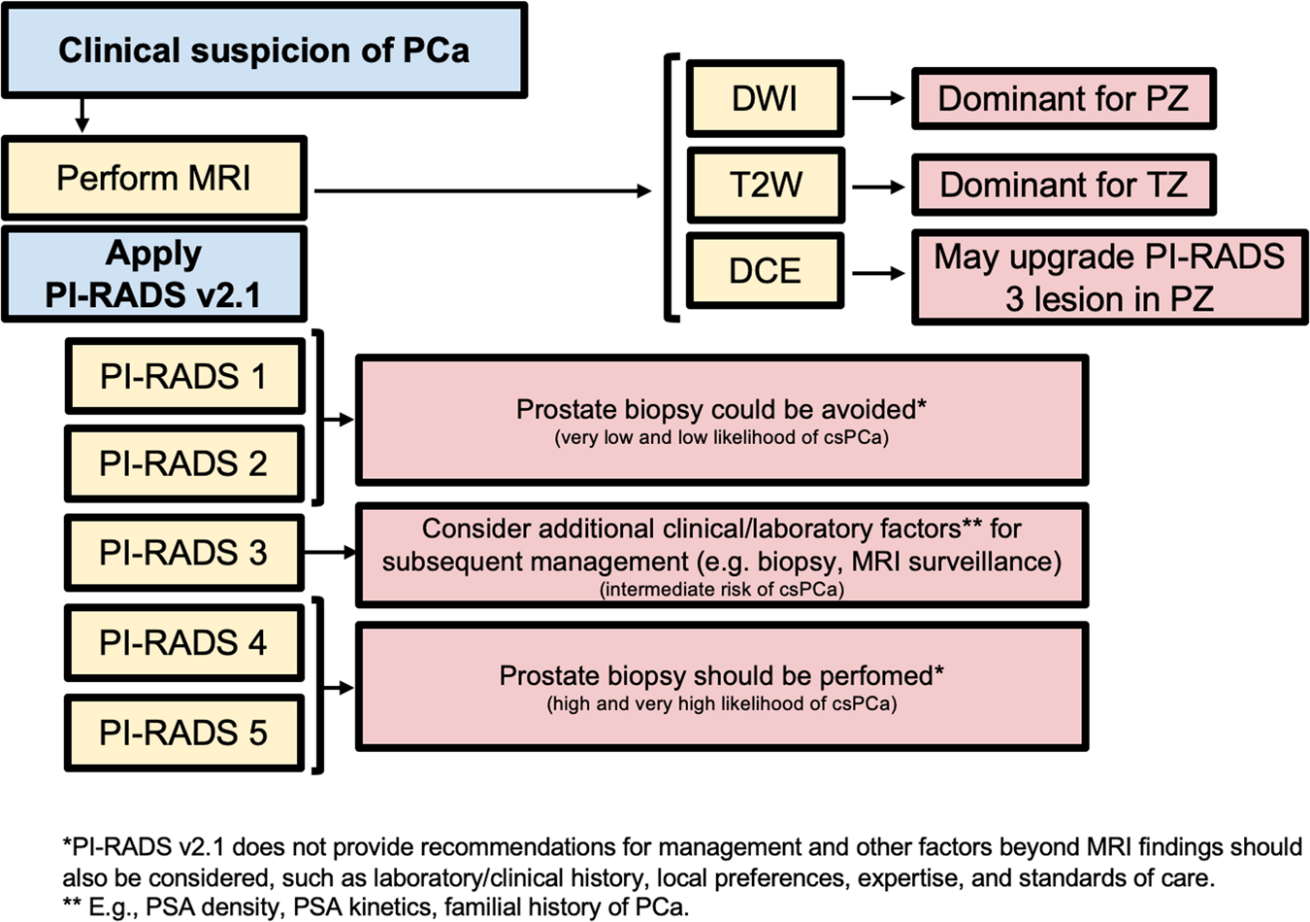
Flowchart for how and when PI-RADS v2.1 should be used, with practical implications. PCa, prostate cancer; MRI, magnetic resonance imaging; PI-RADS, Prostate Imaging Reporting and Data System; DWI, diffusion weighted imaging; T2W, T2-weighted; PZ, peripheral zone; TZ, transitional zone; csPCa, clinically significant PCa; PSA, prostate specific agent

The use of prostate MRI before biopsy reduces the number of unnecessary biopsies, maximizes the detection of clinically significant PCa (csPCa), and decreases overdiagnosis of indolent tumors [6]. MRI is recommended as a triage test before biopsy in men with a clinical suspicion of PCa [7]. Additionally, current guidelines strongly recommend adhering to the PI-RADS recommendations when performing and interpreting MRI [7]. PI-RADS was originally introduced by the European Society of Urogenital Radiology (ESUR) in 2012 and updated in the latest version 2.1, to standardize the acquisition, interpretation, and reporting of multiparametric MRI (mpMRI) of the prostate [2]. PI-RADS should be used for the detection and localization of clinically significant lesions in treatment-naïve men undergoing prostate MRI for suspected PCa (Fig. 2). In PI-RADS v2.1, csPCa is defined as Gleason score ≥ 3 + 4 (ISUP grade group ≥ 2), and/or volume ≥ 0.5 cc, and/or extraprostatic extension (EPE) [2].

**Fig. 3:**
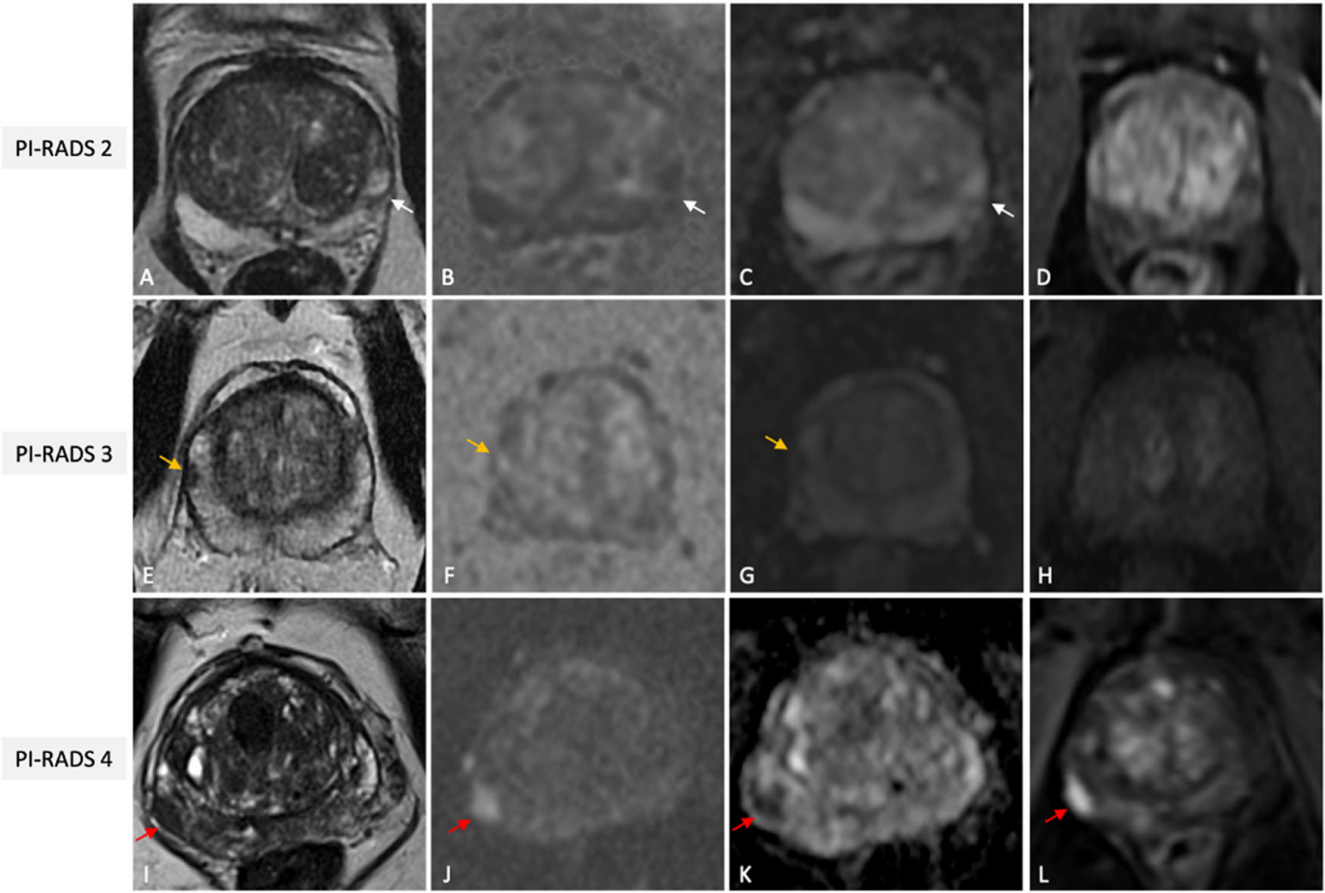
Illustrative examples of PI-RADS scores 2 (**A**–**D**), 3 (**E**–**H**) and 4 (**I**–**L**), from different patients undergoing mpMRI for clinical suspicion of PCa. Wedge shaped area (white arrows) in the left postero-lateral peripheral zone at mid-gland, hypointense on T2w (**A**), slightly hyperintense on synthetic high *b* value DWI (**B**) and hypointense on ADC (**C**) without focal early enhancement on the DCE image (**D**) classified as PI-RADS score 2. Nodular area (orange arrows) in the right anterior peripheral zone at mid-gland, hypointense on T2W (**E**), moderately hyperintense on synthetic high *b* value DWI (**F**) and hypointense on ADC (**G**) without focal early enhancement on the DCE image (**H**), scored as PI-RADS 3. Lenticular shaped area (red arrows) in the right postero-lateral peripheral zone at base, hypointense on T2w (**I**), markedly hyperintense on high *b* value DWI (**J**) and markedly hypointense on ADC (**K**) with focal early enhancement on the DCE image (**L**), scored as PI-RADS 4 with low probability of extra-prostatic extension, then pathologically confirmed via targeted biopsy as clinically significant PCa (GG2). PI-RADS, Prostate Imaging Reporting and Data System; mpMRI, multiparametric magnetic resonance imaging; PCa, prostate cancer; T2W, T2-weighted; DWI, diffusion weighted imaging; ADC, apparent diffusion coefficient; DCE, dynamic contrast enhanced; GG, grade group

The PI-RADS categorical scoring system consists of a 5-point scale representing the likelihood of harboring csPCa for each lesion identified, ranging from “highly unlikely” (PI-RADS 1) to “highly likely” (PI-RADS 5). The PI-RADS score is derived from a combination of prespecified findings on T2-Weighted (T2W), Diffusion-Weighted Imaging (DWI), and Dynamic Contrast-Enhanced (DCE) sequences. The score is zone-specific, meaning that assessments differ for lesions in the peripheral (PZ) and transition zone (TZ). DWI is the dominant sequence when assessing the PZ while T2W is dominant for the TZ. DCE plays a minor role and is used to upgrade (or not) an indeterminate PZ PI-RADS 3 lesion to PI-RADS 4. As an image-based system, clinical factors (such as age, ethnicity, or prostate-specific antigen density (PSAD) value) should not be used in determining the final score. Examples of various PI-RADS scores are shown in Fig. 3. No more than four lesions should be described according to PI-RADS v2.1, and all of them should be reported in a standardized graphical diagram (representing the base, mid-gland, and apex in an axial plane), and explicitly measured (in mm), to standardize MRI review, inter-specialty communication, and targeting of lesions at biopsy.

The role of DCE is debated and non-contrast protocols (often referred to as “biparametric MRI” - bpMRI) may represent a viable alternative to mpMRI [8]. It is important to note that the PI-RADS committee suggests considering bpMRI only for biopsy-naïve individuals who are suspected of having PCa provided that high-quality imaging, expert interpretation, and the ability for patient recall or on-table monitoring are met [9]. Additionally, a clinical risk-based approach may be used to choose between bpMRI and mpMRI [9]. This means conducting bpMRI in lower-risk cases where specificity is prioritized over sensitivity, and mpMRI in higher-risk cases where sensitivity is more crucial.

It is generally accepted that biopsy may be considered for patients with PI-RADS 4–5 and avoided for PI-RADS 1–2, while other clinical factors should be considered for biopsy decisions in patients with PI-RADS 3 lesions. However, it must be noted that PI-RADS v2.1 does not include possible biopsy options, as these must be based on a thorough risk assessment of the patient and strongly depend on local expertise and standard of care [10]. A discussion of biopsy strategies can be found in the PI-RADS MRI-directed biopsy pathway white paper [11].

A meta-analysis reported a pooled sensitivity and specificity of PI-RADS v2.1 of 87% and 74%, respectively, for the detection of Gleason score ≥ 3 + 4 [12]. Of note, the cancer detection rate rises with higher PI-RADS scores, including a step-up function for csPCa in lesions with scores 3–5 [13]. The PI-RADS system’s impact on patient care is substantial [14] allowing its integration into clinical care guidelines [7]. Beyond the diagnostic and management performance, a substantial to excellent reproducibility of the PI-RADS score has been reported [15].

## Prostate Imaging Quality (PI-QUAL) scoring system

**Fig. 4:**
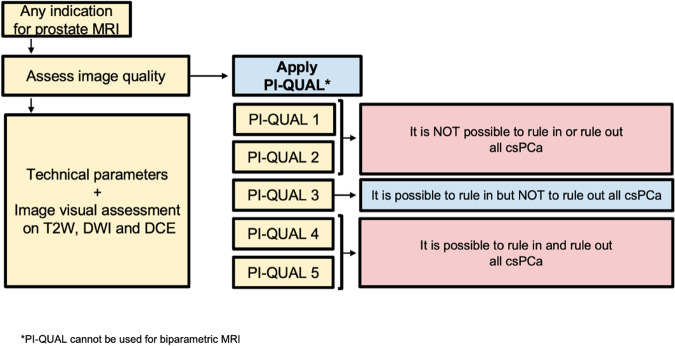
Flowchart for how and when PI-QUAL scoring system should be used, with practical implications. MRI, magnetic resonance imaging; PI-QUAL, Prostate Imaging Quality; T2W, T2-weighted; DWI, diffusion weighted imaging; DCE, dynamic contrast enhanced; csPCa, clinically significant prostate cancer

**Fig. 5:**
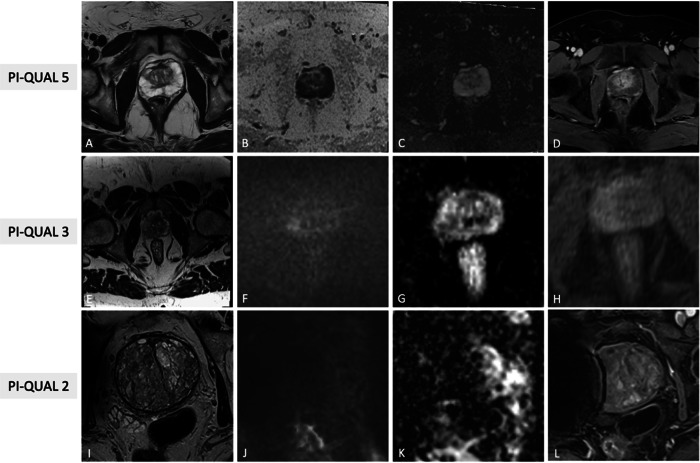
Illustrative examples of PI-QUAL scores 5 (**A**–**D**), 3 (**E**–**H**) and 2 (**I**–**L**) from different patients undergoing mpMRI for various purposes. In the first case, T2W (**A**), synthetic b1500 DWI (**B**), ADC map (**C**) and DCE (**D**) images are of optimal diagnostic quality and meet the technical requirements of PI-RADS v2.1 (PI-QUAL score 5). In the second case, T2W FOV is exceedingly large (**E**), diffusion images are of good quality (**F**, **G**), while DCE shows inadequate in-plan resolution (**H**); since, at least two mpMRI sequences taken together are of diagnostic quality, the exam has been scored as PI-QUAL 3. In the third case, only T2W (**I**) shows optimal quality, while high *b* value DWI (**J**), ADC map (**K**) and DCE (**L**) are suboptimal due to the presence of right hip prostheses (PI-QUAL score 2). PI-QUAL, Prostate Imaging Quality; mpMRI, multiparametric magnetic resonance imaging; T2W, T2-weighted; DWI, diffusion weighted imaging; ADC, apparent diffusion coefficient; DCE, dynamic contrast enhanced; FOV, field of view

Adhering to the technical parameters outlined in the PI-RADS v. 2.1 document is the initial step to ensure the quality of MR images [2]. Nevertheless, even when MRI is performed following these recommendations, patient-related factors (e.g., rectal air, movement, hip prostheses, body habitus) may degrade the quality of the images, thereby affecting the rule-in and rule-out ability of MRI. A consensus document from the ESUR and the European Association of Urology Section of Urological Imaging (ESUI) points out that image quality should be consistently reported in all prostate MRI studies regardless of purpose [16]. To standardize image quality assessment for prostate MRI, the Prostate Imaging Quality (PI-QUAL) scoring system was developed [3]. It consists of a 5-point scale to communicate the reliability of findings based on the image quality (Fig. 4) [17]. Scores of 1 or 2 denote that all or 2 out of 3 sequences fall below the minimum standard, making it impossible to reliably rule-in or rule-out all clinically significant lesions. A score of 3 indicates sufficient diagnostic quality to only rule-in but not rule out lesions, while scores of 4 or 5 indicate that all 3 sequences have sufficient diagnostic quality to both rule-in and rule-out clinically significant lesions. Examples of the various PI-QUAL scores are depicted in Fig. 5.

Promising evidence is emerging on the impact of image quality as assessed by PI-QUAL in various settings, including PCa detection and staging [18, 19], but robust prospective reproducibility and clinical impact studies are needed to assess the broader implications of assessments of image quality. Preliminary data indicate that in a targeted biopsy patient cohort, higher quality PI-QUAL scans (≥ 4) showed significantly higher biopsy yields [18], while for the evaluation of EPE, a scan of low-quality impairs MRI performance compared to clinical staging tools [19].

As for other scoring systems, PI-QUAL is evolving in parallel with clinical experience and the accumulation of scientific evidence. An international working group, from the ESUR and ESUI, is actively collaborating on an updated version to address the current limitations. Specifically, the forthcoming version will streamline the assessment of technical parameters and be applicable for the evaluation of bpMRI. Additionally, assessments of the image quality and of the likely clinical implication are being separated to make the system more applicable to a broader set of MRI applications including AS and population PCa screening.

## Prostate Cancer Radiological Estimation of Change in Sequential Evaluation (PRECISE) scoring system

**Fig. 6:**
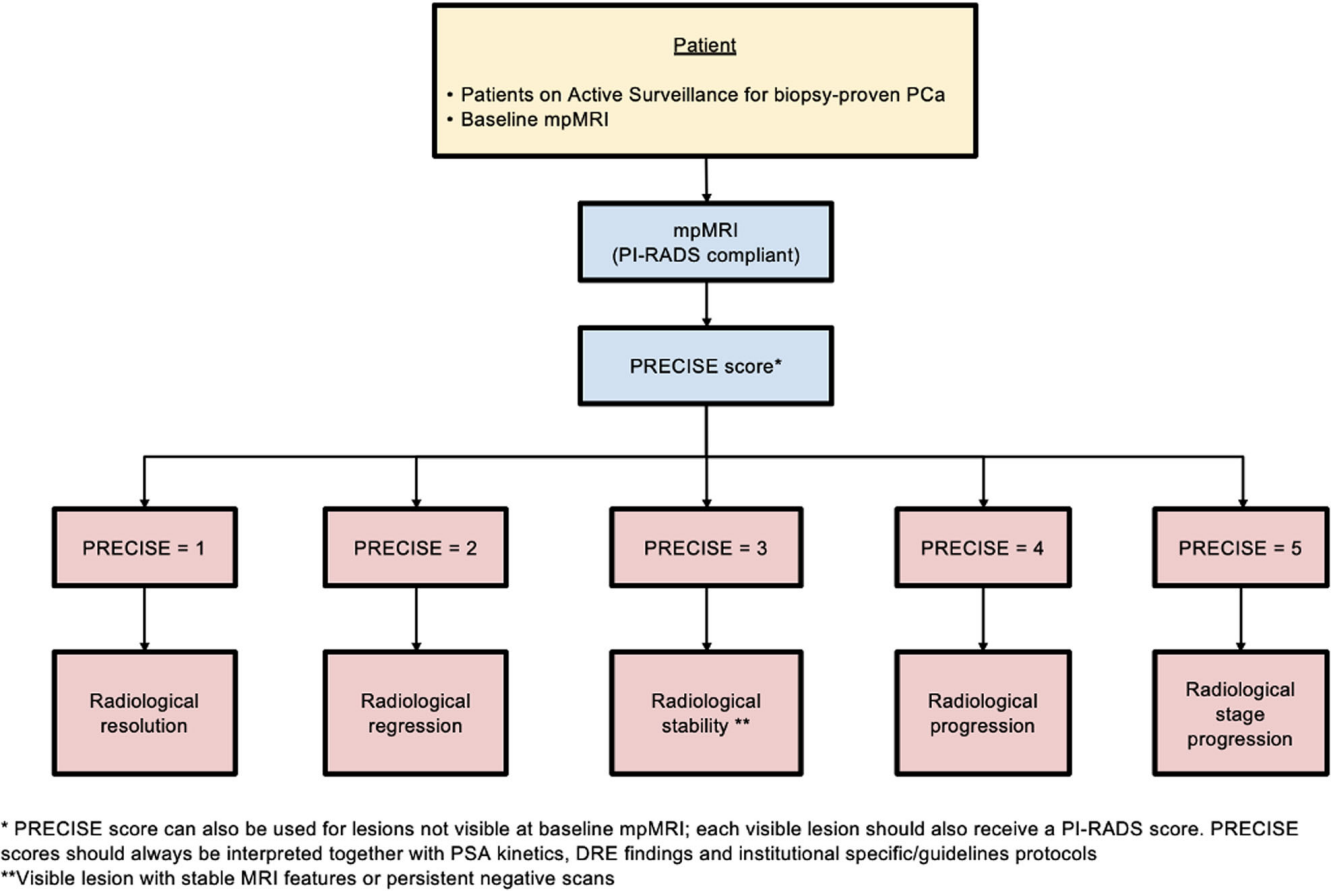
Flowchart for how and when PRECISE scoring system should be used. PCa, prostate cancer; mpMRI, multiparametric magnetic resonance imaging; PI-RADS, Prostate Imaging Reporting and Data System; PRECISE, Prostate Cancer Radiological Estimation of Change in Sequential Evaluation; PSA, prostate specific agent; DRE, digital rectal examination

**Fig. 7:**
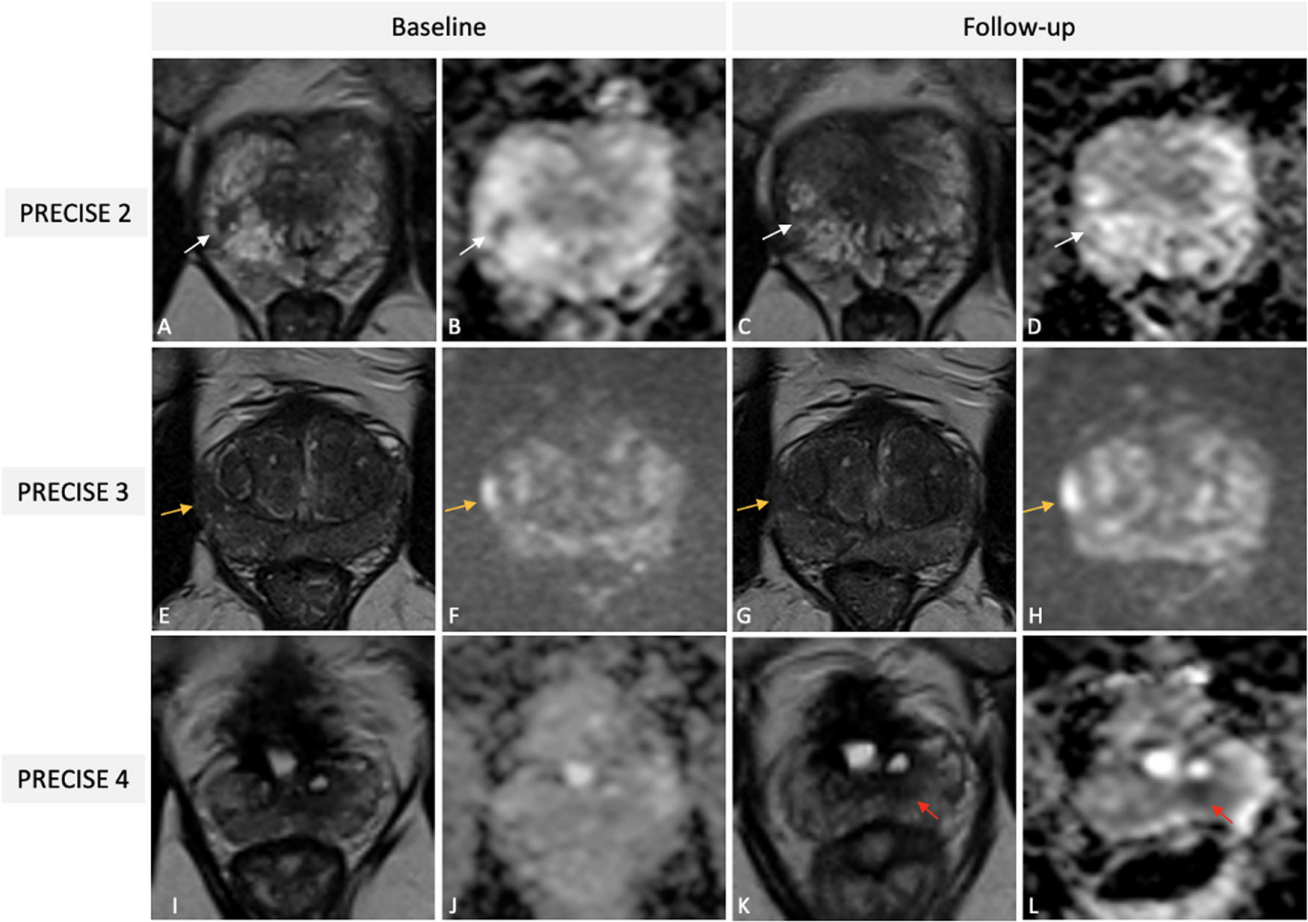
Illustrative examples of PRECISE scores 2 (**A**–**D**), 3 (**E**–**H**), and 4 (**I**–**L**) from different patients on AS for PCa undergoing mpMRI. Baseline T2W (**A**) image and ADC map (**B**) of a 56-year-old patient showing a PI-RADS 4 lesion (white arrows) in the right postero-lateral peripheral zone at the apex, then pathologically confirmed at targeted biopsy as Gleason 3 + 4 PCa (Pattern 4 ≤ 10%). One-year follow-up scan (**C**, **D**) demonstrates a reduction in lesion size on axial T2W (white arrow in **C**) and in conspicuity on ADC map (white arrow in **D**), scored as PRECISE 2 (PI-RADS score 3). Baseline T2w (**E**) and high *b* value DWI (**F**) images of a 69-year-old patient revealing a PI-RADS 4 lesion (orange arrows) in the right postero-lateral peripheral zone at the mid-gland, then pathologically confirmed at targeted biopsy as Gleason 3 + 3 PCa. A three-year follow-up scan (**G**, **H**) indicating stability in both size and conspicuity of the identified lesion, classified as PRECISE score 3 (PI-RADS score 4). Baseline T2w image (**I**) and ADC map (**J**) of a 71-year-old patient, with a previous history of TURP, showing absence of suspicious lesions (PI-RADS score 2); systematic biopsy revealed a Gleason 3 + 3 PCa. A two-year follow up scan showed the presence of a small nodular lesion (red arrows in **K** and **L**) in the left postero-median peripheral zone at the mid-gland and was classified as PRECISE score 4 (PI-RADS score 4); targeted biopsy yielded upgrade to Gleason score 3 + 4. PRECISE, Prostate Cancer Radiological Estimation of Change in Sequential Evaluation; AS, active surveillance; PCa, prostate cancer; mpMRI, multiparametric magnetic resonance imaging; T2W, T2-weighted; ADC, apparent diffusion coefficient; PI-RADS, Prostate Imaging Reporting and Data System; DWI, diffusion weighted imaging; TURP, transurethral resection of the prostate

AS represents a management strategy for indolent PCa that consists of closely monitoring the disease rather than opting for immediate active treatment. Eligibility for AS involves a biopsy Gleason score ≤ 3 + 4, clinical stage ≤ T2b and a serum PSA level < 10 ng/mL, but criteria vary considerably across guidelines and practices [20]. The current guidelines from the European Association of Urology (EAU), American Urological Association (AUA), and UK National Institute for Health and Care Excellence (NICE) uniformly advocate using MRI for patient selection and subsequent AS assessments. To overcome the lack of standardization in MRI reporting, the Prostate Cancer Radiological Estimation of Change in Sequential Evaluation (PRECISE) scoring system was introduced in 2016 to evaluate radiological changes on serial imaging [4]. The PRECISE score takes into account different MRI features, including lesion conspicuity and size, and assigns a score ranging from 1 to 5: a PRECISE score of 1 or 2 implies radiological regression, a PRECISE score of 3 indicates radiological stability, while a PRECISE category of 4 or 5 denotes radiological progression [21]. Figures 6 and 7 provide the definition of each score and a pragmatic application of the system. Using the dedicated case report form, radiologists are able to comment on the three most prominent lesions, assigning higher importance to the “index” intraprostatic lesion. For each lesion, it is imperative to determine its appearance compared to the baseline scan, its current visibility status, and its size, which can be calculated according to different definitions (i.e., single diameter, biaxial diameter, ellipsoid formula or planimetry). For patients with visible target lesions measurements should be done on the dominant sequence as per PI-RADS version 2.1 [21]. Clearly, these assignments can only be done on scans of high quality. Additionally, it is important to emphasize that PI-RADS and PRECISE are not mutually exclusive. Indeed, according to PRECISE when interpreting MRI examinations radiologists should categorize MRI-visible lesions using PI-RADS, in addition to assigning a PRECISE change score. Note that the PRECISE system is not meant to be used for the follow-up of patients with negative biopsies, nor to evaluate treatment response.

There are noteworthy areas of uncertainty in the PRECISE recommendations, particularly in defining imaging changes. Terms like “reduction in volume,” “significant increase,” and “lesion conspicuity” can be subjective, leaving room for discrepancies in clinical interpretations. Also, variability in measurements over time and fluctuations in apparent size can occur [4, 21]. The conspicuity of the lesion may also change with the use of different magnets, field strengths, coils, or vendors, and across centers, potentially affecting the reproducibility of apparent diffusion coefficient (ADC) values (although this latter is not formally included in the current scoring system) from serial scans. Furthermore, alterations in the background, such as inflammation and the development of scarring and cystic atrophy, can also influence the overall assessment. An updated version of the scoring system (PRECISE v. 2) containing clarifications in areas of uncertainty surrounding the use of serial MRI in AS and highlighting areas for further research, has just been published [22].

In a recent meta-analysis, predominantly comprising retrospective studies, PRECISE demonstrated a robust pooled negative predictive value (NPV) of 0.88 (95% CI, 0.81–0.94) but had a lower pooled positive predictive value (PPV) of 0.51 (95% CI, 0.31–0.70) for predicting disease volume or grade progression [23]. Consequently, current evidence suggests that determining the triggers for follow-up and re-staging biopsies should be based on a risk-adjusted use of MRI, prompted by clinical factors and biomarkers, rather than only using imaging changes as indicators for early re-biopsy or treatment initiation [24].

## Prostate Imaging for Local Recurrence Reporting (PI-RR) scoring system

Patients with PCa treated with either radiation therapy (RT) or radical prostatectomy (RP) can experience biochemical recurrence (BCR), or PSA persistence after surgery [25, 26]. Clinical guidelines support the use of both MRI and prostate-specific membrane antigen-positron emission tomography (PSMA-PET) for the detection of local recurrence [7], with MRI being recommended especially in patients experiencing BCR after RT.

**Fig. 8:**
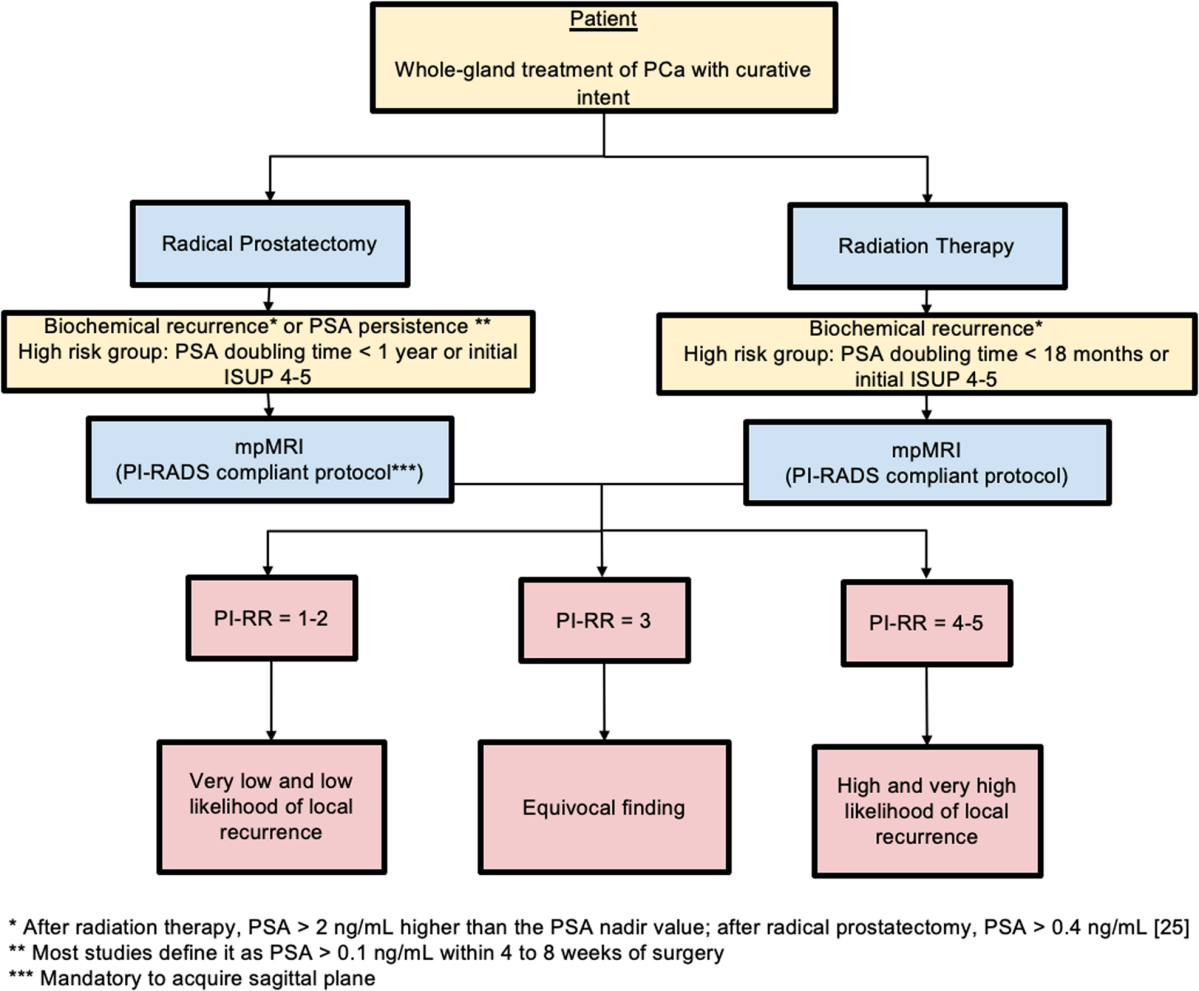
Flowchart for how and when PI-RR scoring system should be used. PCa, prostate cancer; PSA, prostate specific agent; mpMRI, multiparametric magnetic resonance imaging; PI-RADS, Prostate Imaging Reporting and Data System; PI-RR, Prostate Imaging for Local Recurrence Reporting; ISUP, International Society of Urological Pathology

**Fig. 9:**
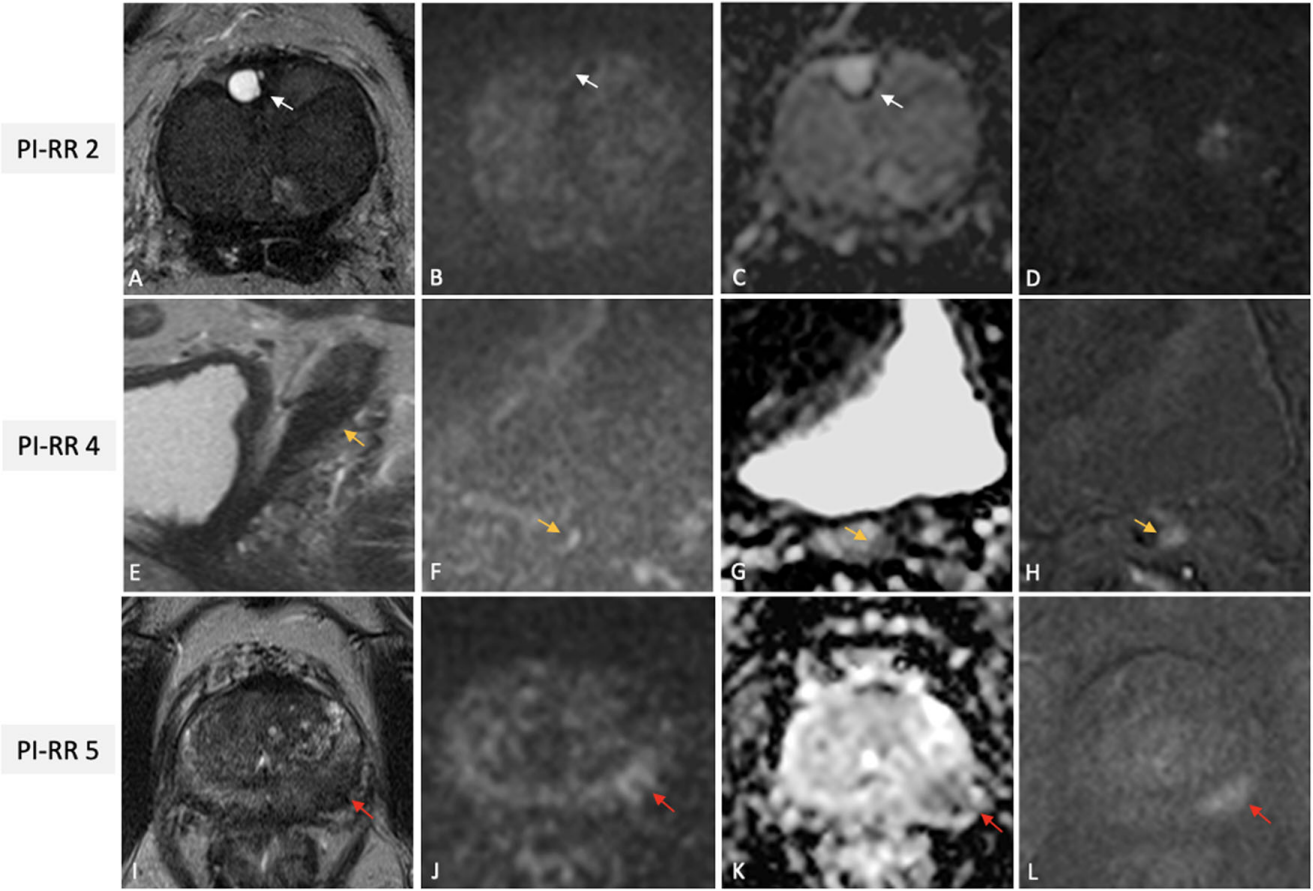
Illustrative examples of PI-RR scores 2 (**A**–**D**), 4 (**E**–**H**) and 5 (**I**–**L**), from different patients undergoing mpMRI for rising PSA values after whole-gland treatment with curative intent for PCa. Images (**A**–**D**) from a 70-year-old man with serum PSA of 0.31 ng/mL after RT for csPCa (GG2) showing a diffusely hypointense gland on axial T2W (**A**), with a focal fluid-filled hyperintense nodule in the right anterior transitional zone at prostate base (white arrow), with no restricted diffusion (with arrows in **B** and **C**) nor early enhancement on the DCE image (**D**), scored as PI-RR 2 (residual cystic atrophy). Images of a 72-year-old man with BCR (PSA value = 0.81 ng/mL) after RP for PCa (GG1), showing a masslike (orange arrows) hypointense focus on sagittal T2W, at the origin of the seminal vesicle residues, with focal marked hyperintensity on high–*b* value DWI (**F**), hypointense on ADC (**G**) and with focal early enhancement on the DCE image (**H**); the case was scored as PI-RR 4 (no data on primary tumor side). Images (**I**–**L**) of a 64-year-old man with BCR (PSA value = 3.4 ng/mL) 2 years after RT for csPCa (GG2) showing a masslike focus on the left postero-lateral peripheral zone at mid-gland (red arrows) hypointense on axial T2W (**I**) with focal marked hyperintensity on high–*b* value DWI (**J**), hypointense on ADC (**K**) with focal early enhancement on the DCE image (**L**) at the same site of the primary tumor, scored as PI-RR 5. PI-RR, Prostate Imaging for Local Recurrence Reporting; mpMRI, multiparametric magnetic resonance imaging; PSA, prostate specific agent; PCa, prostate cancer; RT, radiation therapy; csPCa, clinically significant PCa; GG, grade group; T2W, T2-weighted; DCE, dynamic contrast enhanced; BCR, biochemical recurrence; RP, radical prostatectomy; DWI, diffusion weighted imaging; ADC, apparent diffusion coefficient

The Prostate Imaging for Local Recurrence Reporting (PI-RR) scoring system was developed to standardize acquisition, interpretation, and reporting of pelvic MRI in patients after whole-gland therapies done with curative intent [5]. PI-RR is a 5-point assessment scale defining the likelihood of local recurrence from “very low” to “very high” (Fig. 8). MR images should be acquired using the PI-RADS v2.1 technical recommendations, noting that after RP sagittal planes should be always acquired. The reporting criteria are based on anatomical and functional imaging findings. T2W images are used to locate suspicious lesions and to compare them to preoperative imaging, but do not take part in the final assessments. DWI and DCE are both considered as the codominant sequences in patients treated with RT. After RP, DCE is the dominant sequence, and its quality is of the utmost importance. Consequently, a bpMRI protocol in this setting should not be used. After both RP and RT, PI-RR scores of 1 and 2 are assigned when no abnormalities are detected or when “benign” findings are identified, such as fibrotic tissue or residual benign prostatic hyperplasia nodules (Fig. 9A–D). PI-RR scores 4 or 5 should be assigned according to primary tumor location (Fig. 9E–L). A score of 5 should be given when the lesion occurs at the primary tumor site; alternatively, a score of 4 applies if the finding appears in a different location or when the primary tumor location is unknown.

The PI-RR scoring system has been retrospectively validated showing high performance in detecting local recurrence, with moderate to high inter-reader agreement [27]; however, multicenter prospective studies are still needed. The primary drawback of the PI-RR system is that it assesses the relapse risk only within the prostate gland or prostatic bed after whole-gland therapies. Additionally, the clinical adoption of PI-RR is impacted by the ongoing clinical paradigm shift in the imaging workup of BCR through the preferred clinical adoption of ^68^Ga- and ^18^F-PSMA-PET [7] after RP.

Recently, two scoring systems have been proposed to evaluate the likelihood of residual/recurrent disease after focal therapy (Prostate Imaging after Focal Ablation -PI-FAB- and the Trans-Atlantic Recommendations for prostate Gland Evaluation with MRI after focal Therapy-TARGET) but both systems lack clinical validation [28, 29]. Additionally, it is important to recognize the presence in scientific literature of other, albeit less commonly employed tools for PCa assessments [30], such as the grading system for predicting EPE [30].

## Summary statement

The incorporation of clinically relevant scoring systems like PI-RADS, PI-QUAL, PRECISE, and PI-RR reflects ongoing endeavors to standardize image reporting, promote validation research and improve communication. Given the pivotal role radiologists play in different phases of PCa diagnosis and management, a comprehensive understanding of these scoring systems and their appropriate application is essential for enhancing clinical utility. However, it should be noted that these scoring systems are evolving with ongoing refinements and adaptations to emerging clinical insights and scientific evidence.

## Patient summary

Radiologists should have a good understanding of prostate MRI-based scoring systems and their application in the various clinical scenarios of PCa management. Standardized reporting enhances the practicality of MRI in clinical settings, facilitating its integration into routine practice with the aim of improving patient outcomes.

## Abbreviations

AS: Active surveillance
csPCa: Clinically significant prostate cancer
EPE: Extraprostatic extension
mpMRI: Multiparametric MRI
PCa: Prostate cancer
PI-QUAL: Prostate Imaging Quality
PI-RADS: Prostate Imaging Reporting and Data System
PI-RR: Prostate Imaging for Local Recurrence Reporting
PRECISE: Prostate Cancer Radiological Estimation of Change in Sequential Evaluation
